# Quantitative Benefit of Inpatient Dermatology Services on Hospital Length of Stay in an Academic Hospital

**DOI:** 10.7759/cureus.43519

**Published:** 2023-08-15

**Authors:** Camilla Reimer, Erica Lee, Ashley Wysong, Corey Georgesen

**Affiliations:** 1 Department of Dermatology, University of Nebraska Medical Center, Omaha, USA

**Keywords:** dermatology consult, cutaneous disease, quality of care, care outcomes, inpatient consults, hospital dermatology

## Abstract

Background: Dermatologic disease has been shown to have high rates of diagnostic and treatment discordance between dermatologists and non-specialists. Inpatient dermatology consultative services have the potential to improve patient care, but there is a paucity of data evaluating the quantitative effects of such services. This study aimed to evaluate the impact a newly established inpatient dermatology service had on quantitative patient care outcomes.

Methods: This retrospective cohort study compared quantitative care measures of dermatologic inpatients during the years both pre- and post-implementation of an academic hospital’s dermatology consultative service. The primary outcomes included hospitalization duration, readmission rates, and establishment of outpatient dermatologic care.

Results: The study found a 1.04-day reduction in hospital length of stay (p-value = 0.046) after the consultation service establishment. Additionally, there was a significant increase in the rate by which patients sought outpatient dermatology follow-up (6.7% versus 24.4%, p-value <0.001). No significant change in the all-cause readmission rate was identified.

Conclusion: The reduction of hospitalization duration supports inpatient dermatology services as a viable means to provide improved patient care and reduce health systems costs. Hospitals that do not have a consulting service for cutaneous conditions provided by a dermatology specialist should strongly consider establishing such a department.

## Introduction

With over 600,000 hospital admissions per year in the United States alone, cutaneous conditions create a large burden for our healthcare system [1-3]. Prior literature has emphasized the importance of hospital dermatologists in providing accurate diagnosis and disease treatment. Because of the difficulty in differentiating and treating conditions of the skin without specialist training, these studies have shown a 45% to 76% diagnostic discordance and 72% to 97% treatment discordance between dermatologists and non-dermatologists [2,4-9]. These high rates of misdiagnosis and treatment provide qualitative support for the benefit of the direct involvement of dermatologists in skin pathologies.

There is a paucity of data that analyzes hospital dermatologists’ quantitative patient care outcomes. Only a few studies have sought to elicit these quantitative effects but have returned with mixed results. In evaluating hospital length of stay, three different studies each found consultation to be associated with an increase, a decrease, and no change in hospital duration [2,6,10]. Another study looked into a same-cause readmission rate and found a 10-fold decrease in odds of readmission, but this result has not been replicated [10].

The aim of this study was to further evaluate the quantitative effects inpatient dermatology consultative services have on patient care outcomes. Based on previous research’s qualitative support of consultations, it was hypothesized that there would be a reduction in one-year hospital readmission rates and shorter length of hospitalization in the year following the establishment of the consult service.

## Materials and methods

A retrospective cohort review study of 159 electronic medical records was conducted among patients who were admitted to a tertiary care academic hospital in Omaha, Nebraska. This hospital implemented its inpatient dermatology consultation service in October 2018. Inclusion in the study necessitated a cutaneous condition to be the primary discharge diagnosis, as specified by the ICD-10 codes of common dermatologic conditions outlined in Table 1 [4,6,8,11-13]. To compare cohorts of patients during the years immediately preceding and proceeding service establishment, hospital admission was required during the years of 2017 or 2019.

**Table 1:** Diagnostic groups and ICD-10 codes for inclusion criteria

Diagnostic Groups and Specific Diagnoses	ICD-10 Codes for Inclusion
Viral Infections Characterized by Skin and Mucous Membrane Lesions	B02
Purpura and other Hemorrhagic Conditions	D69
Infections of the Skin and Subcutaneous Tissue	L03
Dermatitis and Eczema	L20, L21, L23, L24, L25, L26, L28, L29, L30
Urticaria and Erythema	L50, L51, L52, L53
Disorders of Skin Appendages	L72, L73
Systemic Connective Tissue Disorders	M31.0
Other Skin Symptoms and Conditions	B35, L82, L88, L94, L95, L98, Q81, R21
Cutaneous T-cell lymphoma	C84.A
Drug Rash with Eosinophilia and Systemic Symptoms	D72.12
Graft-versus-host Disease	D89.81

Data were collected through Nebraska Medicine’s Electronic Health Record Data Access Core service and included patient demographics, hospital diagnoses, length of stay, readmission dates and diagnoses, and post-discharge outpatient dermatology visits. The study protocol was approved by Nebraska Medicine’s International Review Board (approval number 0338-20-EP).

Of the 159 charts reviewed, five were excluded due to extensively prolonged hospitalizations for unrelated disposition problems. The pre-service implementation group of 2017 admissions served as the control group (n=80), as they did not have access to dermatology specialists. The 2019 post-implementation cohort served as the exposure group (n=74) since these patients had access to consulting services. Statistical analysis using independent t-tests was performed to evaluate the length of stay, all-cause and dermatologic-cause one-year readmissions and outpatient dermatology follow-up. Demographic comparability between the groups was also performed with independent t-tests. Additionally, linear regression was performed in the analysis of the demographic covariate effect on the primary length of stay outcome.

## Results

Descriptive statistics of group demographics are outlined in Table 2. All demographics were comparable between the two groups besides a higher percentage of Hispanic and other ethnicities among the pre-implementation group (p = 0.01). Linear regression of these demographics showed no significant effect on the hospital length of stay outcome (Table 3).

**Table 2:** Patient characteristics and demographics

Characteristic	2019/Exposure Group, (n=74)	2017/Control Group, (n=80)	P-value
Male sex, No. (%)	35 (47)	33 (41)	0.23
Age, mean years	50.9	46.9	0.12
BMI, mean	30.2	29.0	0.23
Ethnicity, No. (%)			
Non-Hispanic	72 (97)	70 (88)	0.01
Hispanic	2 (3)	10 (13)	0.01
Race, No. (%)			
White	61 (82)	59 (74)	0.10
Black/African American	10 (14)	10 (13)	0.43
American Indian/Alaska Native	2 (3)	3 (4)	0.36
Other	1 (1)	8 (10)	0.01

 

**Table 3:** Statistical significance of patient demographics effect on hospital length of stay

Characteristic	Effect on Length of Stay (Regression p-value)
Male sex	0.22
Age	0.56
BMI	0.57
Ethnicity	
Non-Hispanic	0.35
Hispanic	0.35
Race	
White	0.30
Black/African American	0.53
American Indian/ Alaska Native	0.20
Other	0.891
Diagnosis Category	
Viral Infections	0.15
Purpura and Other Hemorrhagic Conditions	0.07
Infections of the Skin	0.17
Dermatitis and Eczema	0.73
Urticaria and Erythema	0.19
Disorders of Skin Appendages	0.54
Systemic Connective Tissue Disorders	0.23
Other Skin Symptoms	0.71

The post-service implementation group showcased a 1.04-day reduction in hospital length of stay, with a mean of 4.27 days in the 2017 control group and 3.23 days in the 2019 exposure (p = 0.046). The exposure group also exhibited an increase in the percentage of patients who sought follow-up dermatology outpatient care, increasing from 6.2% in 2017 to 24.4% in 2019 (p < 0.001) (Table 4). No significant difference was identified for all-cause or dermatologic-cause hospital readmissions (Table 5). Among the 2019 exposure group, 25.7% received an inpatient consult, while the other 74.3% did not.

**Table 4:** Post-discharge outpatient dermatology visit

	2019 Exposure Group (n = 74)	2017 Control Group (n = 80)	P-value
Post-discharge Outpatient Dermatology Visit, % (No.)	24.4 (18)	6.2 (5)	0.0008
Outpatient Visit < 60 days Post-discharge, % (No.)	13 (17.6)	0 (0)	0.00003

**Table 5:** Rate of one-year readmission

	2019 Average Rate (95% CI)	2017 Average Rate (95% CI)	Difference in Rate	P-value
All Cause Readmission	0.365 (0.254-0.475)	0.388 (0.280-0.495)	-0.023	0.387
All Dermatology Specific Readmission	0.081 (0.018-0.144)	0.063 (0.009-0.116)	0.019	0.329

## Discussion

Cutaneous conditions are notoriously difficult to accurately diagnose and treat without specialized training [2,5,9]. Prior studies have highlighted the high rates of discordance in diagnosis and treatment between dermatologists and non-dermatologists [2,4-9]. While providing strong qualitative support for the benefit of the direct involvement of dermatologists in care for cutaneous conditions, many of these studies faced limitations due to the nature of their primary outcomes and comparison groups. Using quantitative primary outcomes and similar cohorts for direct comparison groups, the current study was able to avoid the limitations of previous research.

Most prior studies have utilized diagnostic or treatment discordance between dermatologists and non-dermatologists as a primary outcome, submitting to the assumption that the dermatologists’ assessment and plan are invariably accurate [9]. The numerical care outcomes evaluated in this study allowed for the avoidance of subjective assumption of care benefit and provided information on the concrete magnitude of the service’s impact. Additionally, prior studies have compared patients who received a consultation to those who did not, which lends itself to a selection bias with disease severity [6]. Requests for consultations have been shown to be made in only a fraction of conditions, with a large variance in consult requests based on disease severity and etiology [6]. Comparison based on consultation status, therefore, could mask the benefits of consultation due to the predisposition of poorer outcomes portended by severe disease [6]. This selection bias likely contributes to the conflicting and inconclusive results of hospital dermatologists’ effect on length of stay [2,6,10]. This study avoided the severity selection bias by comparing the sum of all dermatologic patients admitted during one calendar year to the sum of another calendar year, permitting greater similarity between cohorts than in groupings of consulted versus non-consulted patients. Additionally, because this study did not focus on only those individuals who received consultations, it was able to assess the impacts made across the whole population of dermatologic patients, and therefore showcase any large-scale benefits consultative services brought to the hospital.

Of key importance, this study found a significant 1.04-day reduction in hospital length of stay among the post-implementation cohort. The association between reduced length of mean hospital stay and availability of dermatologic consults provides support for the efficacy and necessity of these services in the inpatient setting. However, less than 25% of patients in this group received a consult.

A possible explanation for this significant reduction despite the low consultation rate is that consults placed for the most severely diseased allowed for large outcome improvement among these patients. In turn, the mean measures of care for the whole population improved. Regardless of the mechanism of improvement, there was still a tangible benefit of having the consultative service, even with low rates of consultation. Therefore, it is likely that with higher utilization of the service, there may be even greater improvements in care measures.

Of note, there was a significant increase in the rate of seeking specialized outpatient follow-up care after the service’s establishment. The increase from 6.2% to 24.4% of patients is likely due to patient knowledge of the existence of specialists, as well as dermatologist-directed education about the importance of specialized care. While this numerical increase does not directly measure care quality, it can be hypothesized that patients who receive specialized follow-up will have comprehensive disease treatment, potentially preventing same-cause readmission. Unfortunately, there was no significant difference found in hospital readmission rates. Despite prior studies finding a 10-fold decrease in readmission [10], the limited number of readmissions, especially those from a dermatologic cause, did not have the power to observe any significant difference between the groups. Future studies with larger sample sizes and numbers of readmissions could be beneficial in further investigating this care outcome.

While overcoming the limitations of prior research, this study does have a few important limitations of its own. In addition to its limited sample size, the study’s location at a single midwestern academic hospital may not allow for generalizability to all hospital settings. Finally, the nature of chart review introduces the potential for variability in documentation, specifically in ICD-10 code selection, which could lead to inaccuracies in data.

## Conclusions

The results of this study showed that the establishment of dermatologic consultative services in an inpatient setting resulted in a significant reduction in hospital length of stay among dermatologic patients, as well as increased rates of patients seeking specialist outpatient follow-up. The reduction in hospital length of stay is an indicator of more efficient care that may also reduce the overall financial burden. This study took on a new perspective in its analysis by evaluating the totality of the dermatologic patient population instead of comparison based on consultation status, which allowed for the determination of the effect that the availability of the service has on overall patient care.

The benefit of service availability was indicated by the reduced hospitalization length and was present despite the low rate of consultation among the post-implementation cohort. Higher utilization of consultations may result in greater improvements in quality-of-care measures. The quantitative improvement in shorter hospital stays adds to the previously established qualitative benefit of hospital dermatologists in appropriate diagnosis and treatment. Hospitals should consider the implementation and utilization of inpatient dermatology consultative services to improve patient care for cutaneous disease.
